# 1306. Lower Treatment Failure Rates in Patients with Non-Staphylococcal Prosthetic Joint Infections (PJI) Treated with Debridement and Implant Retention (DAIR) Receiving Chronic Antibiotic Suppression (CAS)

**DOI:** 10.1093/ofid/ofad500.1145

**Published:** 2023-11-27

**Authors:** Poorani Sekar, James Merchant, Bruce Alexander, Kelly Miell, Brice Beck, Rajeshwari Nair, Daniel Suh, Christopher Richards, Mireia Puig-Asensio, Andrew Pugely, Julia Walhof, Kimberly Dukes, Marin Schweizer

**Affiliations:** University of Iowa Hospitals and Clinics, Iowa City, Iowa; Iowa City VA Health Care System, Iowa City, Iowa; Iowa City VA Medical Center, Iowa City, Iowa; Iowa City VA Health Care System, Iowa City, Iowa; Iowa City VA Health Care System, Iowa City, Iowa; University of Iowa, Iowa, Iowa; Iowa City VA Health Care System, Iowa City, Iowa; Iowa City VA Health Care System, Iowa City, Iowa; University of Iowa, Iowa, Iowa; University of Iowa Hospital and Clinics, Iowa City, Iowa; Iowa City VA Health Care System, Iowa City, Iowa; University of Iowa Carver College of Medicine, Iowa City, Iowa; William S. Middleton VA Hospital, madison, Wisconsin

## Abstract

**Background:**

PJI occurs in 0.5-2% of Joint Arthroplasty. Patients with acute non-Staphylococcal PJI who undergo DAIR are treated with 6 weeks of antimicrobials after which CAS may be considered. We aimed to compare the incidence of treatment failure between people who received CAS and those who did not.

**Methods:**

This is a retrospective cohort study of patients admitted to Veterans Affairs (VA) hospitals from 2003-2017 with a non-Staphylococcal PJI, underwent DAIR and received 6 weeks of antimicrobial treatment (Table 1). CAS was defined as at least 30 days of oral antibiotics after 6 weeks of antimicrobial treatment. Duration of CAS was categorized as short (1-3 months), moderate (3-6 months) and long ( >6 months) (Fig 1). Patients were followed for 5 years. Treatment failure was defined as microbiologically confirmed recurrent PJI, additional debridement or re-operation at the same site. Cause-specific Kaplan-Meier curves were used to compare treatment failure rates between those who did and did not receive CAS, censoring on death.

**Figure:**
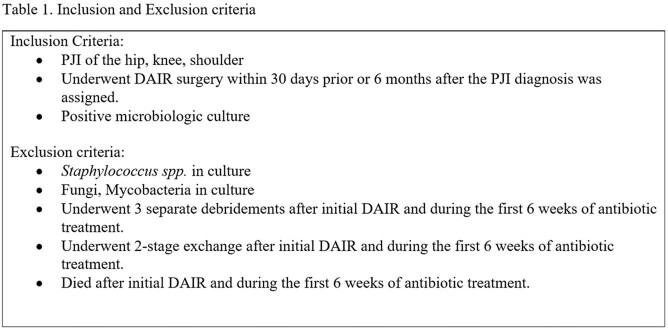

**Figure:**
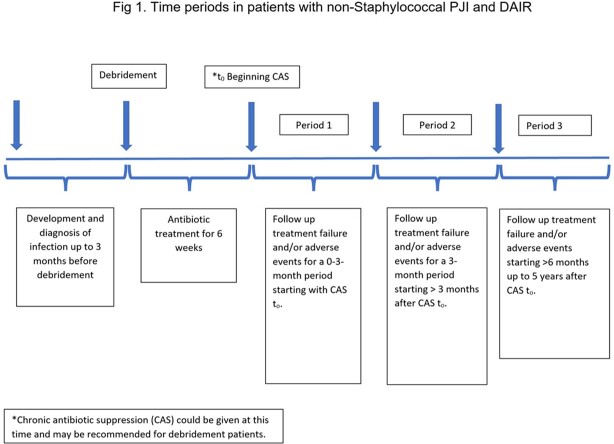

**Results:**

Among 468 patients with non-staphylococcal PJI who underwent DAIR, 208 (44.4%) received CAS. Patients with Enterococcus PJI were statistically more likely to receive CAS. K-M curves showed patients on CAS had a higher estimated failure free survival probability at 5 years when compared to those who did not get CAS (66% vs. 55%, p< 0.01) (Fig 2). When antibiotic use was considered as a time-dependent covariate, CAS was associated with a decreased hazard of treatment failure (hazard ratio (HR): .47 (95% confidence interval [CI]: 0.29, 0.76). After statistically adjusting for surgical site, severity of illness, and alcohol abuse, a short duration of CAS was significantly associated with decreased treatment failure (HR=0.24; 95% CI: 0.11, 0.52). There was no significant association between moderate or long duration of CAS and treatment failure (Table 2)

**Figure:**
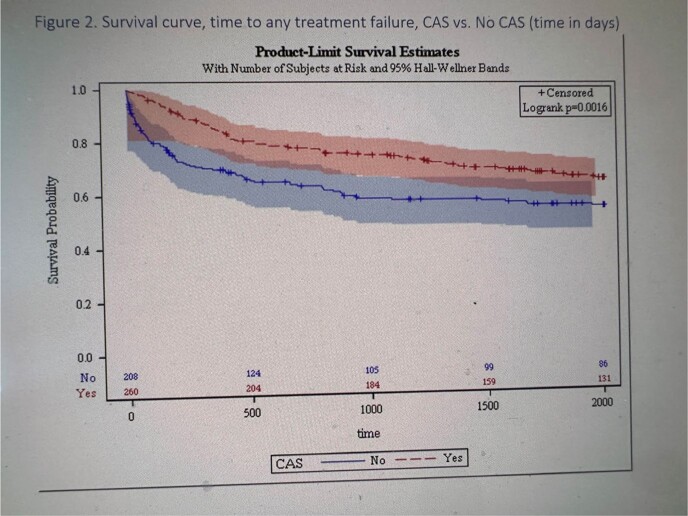

**Table 2**

**Figure:**
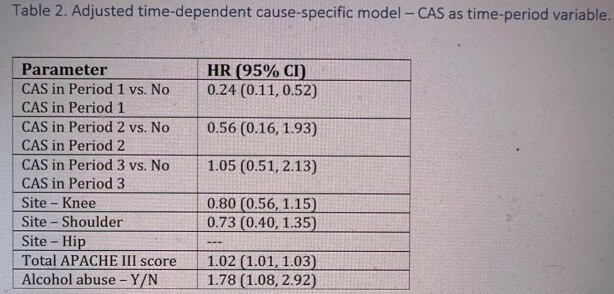

**Conclusion:**

A short duration of CAS may be beneficial among patients with non-Staphylococcal PJI who underwent DAIR. However, there was not a statistically significant association between longer duration of CAS use and treatment failure. Thus, the risks and benefits of long-term antibiotics should be weighed when aiming to prevent recurrence of PJI.

**Disclosures:**

**Mireia Puig-Asensio, MD**, GILEAD: Honoraria **Andrew Pugely, MD, MBA**, Globus Medical: Advisor/Consultant|Globus Medical: Grant/Research Support|Globus Medical: IP royalties|Medtronic: Advisor/Consultant|Medtronic: Grant/Research Support|RDB Bioinformatics: Grant/Research Support|United Healthcare: Advisor/Consultant

